# Mechanical Behavior and Surface Properties of Silicone and PVC Gastric Tubes Under Controlled pH Conditions

**DOI:** 10.3390/ma19153158

**Published:** 2026-07-23

**Authors:** Maja Adamiec, Julia Brodniewicz, Paulina Ciołek, Sylwia Łagan

**Affiliations:** Faculty of Mechanical Engineering, Department of Applied Mechanics and Biomechanics, Cracow University of Technology, Warszawska 24, 31-155 Kraków, Poland

**Keywords:** gastric feeding tubes, silicone, poly(vinyl chloride), wettability, surface free energy, mechanical properties, in vitro aging

## Abstract

In this study, the mechanical behavior, wettability, and surface free energy (SFE) of gastric feeding tubes made of silicone and poly(vinyl chloride) (PVC) were investigated under controlled pH conditions and varying exposure times. Wettability was evaluated using contact angle measurements, and SFE was determined using the Owens–Wendt–Rabel–Kaelble (OWRK), Wu, and van Oss–Chaudhury–Good (vOCG) approaches. Mechanical behavior was analyzed by static tensile testing and stopper pull-off tests. The results showed distinct differences between the materials. PVC tended to exhibit a more stable mechanical response under the tested conditions, whereas silicone exhibited lower stiffness and greater sensitivity to environmental exposure. Surface analysis indicated that PVC maintained relatively stable SFE values (~41–45 mJ/m^2^ according to the OWRK model), whereas silicone exhibited more pronounced variations (~19–34 mJ/m^2^), primarily associated with changes in polar surface components. These modifications became more pronounced with prolonged incubation and occurred alongside changes observed in mechanical response. The findings provide insight into the long-term performance of gastric feeding tube materials and highlight the importance of considering both mechanical and surface properties during material evaluation.

## 1. Introduction

According to the U.S. Food and Drug Administration (FDA), a gastrointestinal tube and accessories are defined as devices consisting of flexible or semi-rigid tubing intended for the instillation or withdrawal of fluids, splinting, or control of bleeding within the alimentary tract. This generic device category includes, among others, feeding tubes, nasogastric tubes, Levine tubes, and gastroenterostomy tubes. Within this regulatory framework, the generic device type is primarily classified as Class II (special controls) [1]. A gastric (stomach) tube is used to access the stomach or other sections of the gastrointestinal tract for the administration of nutrition or medication, or for the removal of gastric contents. Gastric feeding tubes can be classified into nasogastric, orogastric, and enteral tubes, and are typically manufactured from materials such as silicone or plasticized PVC. Their duration of use ranges from short-term to long-term applications, depending on clinical requirements [2,3]. The performance and functionality of these devices depend on multiple factors, including material properties, structural design, and clinical handling conditions [3]. Under Regulation (EU) 2017/745 (MDR), devices such as gastric, enteral, or nasogastric tubes are classified according to general rule-based criteria. Their classification depends on the intended purpose and device characteristics, including the route of access (e.g., via body orifices), degree of invasiveness, and duration of use [4].

The mechanical and surface properties of commonly used materials, such as silicone and plasticized PVC, have been widely described in the literature; however, their behavior under physiological conditions remains insufficiently understood [5,6]. The use of gastric feeding tubes is associated with a range of potential complications that may affect patient safety and comfort [2,7,8,9]. It has been reported that there is no universally accepted method for confirming feeding tube position and that further research is needed in this area [10]. These include difficulties during insertion, such as improper placement or mechanical irritation of the mucosa, as well as complications during removal, including increased resistance, discomfort, and risk of tissue damage. Long-term use may also result in tube deformation, blockage, or changes in material properties due to exposure to the biological environment. Therefore, these properties of feeding tubes are crucial for minimizing clinical risks and ensuring reliable device performance. Surface properties, including wettability and surface free energy, are critical determinants of the interaction between feeding tube materials and the biological environment, influencing bacterial adhesion and subsequent biofilm formation [11,12]. Such microbial colonization has been reported on enteral feeding tubes and may contribute to device-related complications [13,14]. Mechanical testing of gastric feeding tubes is essential for evaluating their safety and functionality under clinically relevant conditions. During use, these devices are exposed to continuous mechanical stresses and variable physiological environments, including fluctuations in pH, which may influence their structural integrity. Previous studies have shown that exposure to simulated gastric conditions may alter both the surface characteristics and mechanical properties of nasogastric feeding tubes [15]. The assessment of mechanical properties provides insight into material durability, flexibility, and resistance to degradation. Furthermore, combining mechanical and surface analyses allows differentiation between bulk material changes and surface-related phenomena.

In practice, no single standard is specifically dedicated to gastric feeding tubes; therefore, a combination of standards related to medical devices, materials, and testing methodologies is typically applied. Key standards include ISO 20695:2020 Enteral feeding systems—Design and testing [16], which specifies requirements for enteral feeding systems, including enteral feeding catheters and accessories. In addition, ISO 80369-3 Small-bore connectors for liquids and gases in healthcare applications—Part 3: Connectors for enteral applications [17] is relevant to connector compatibility and connection safety in enteral feeding systems. Other relevant standards include ISO 10993, Biological evaluation of medical devices [18]; ISO 527, Plastics—Determination of tensile properties [19]; ISO 62, Water absorption of plastics [20]; and ISO 8296/ISO 19403, for contact angle and surface free energy measurements [21,22].

Research on feeding tubes is becoming increasingly important, as reflected by the growing number of publications focused on improving treatment quality [7] and patient safety [8]. Moreover, advances in medical technology further contribute to the increasing interest in this field [9]. Despite extensive studies on individual material properties, limited attention has been given to the combined evaluation of mechanical and surface characteristics under varying pH conditions. The aim of this study was to evaluate the effect of controlled pH environments on the mechanical and surface properties of gastric feeding tubes. Particular attention was given to mechanical behavior under static tensile loading and the characterization of surface wettability and surface free energy to assess material performance under clinically relevant conditions.

## 2. Materials and Methods

### 2.1. Sample Preparation and Aging Conditions

The study involved two types of gastric feeding tubes (CH16, 5.28 mm) manufactured from poly(vinyl chloride) (GALMED, Bydgoszcz, Poland) and silicone (Fortune Medical Instrument Corp., New Taipei, Taiwan). From each material, 20 specimens with a length of 120 mm were prepared. Two specimens were retained as reference samples, while the remaining 18 specimens were divided equally among the three incubation media (pH 2, pH 6, and pH 8.5; *n* = 6 per medium). The allocation of specimens is summarized in Table 1.

To investigate the effect of pH on material behavior, three incubation media with different pH levels were prepared: acidic (pH 2), near-neutral (pH 6), and alkaline (pH 8.5). Although gastric fluids are typically acidic, elevated pH values may occur during the administration of medications, enteral nutrition formulations, or in patients receiving acid-suppressive therapy. pH 2 was selected to represent a strongly acidic gastric environment, whereas pH 6 served as a near-neutral reference environment. The alkaline medium (pH 8.5) was included as a clinically relevant condition and as an accelerated exposure environment. The incubation media were replaced weekly to minimize potential pH variations throughout the study. The acidic medium was prepared using a diluted 6% acetic acid solution (commercial vinegar), and the pH was verified using pH indicator strips. The near-neutral medium consisted of physiological saline solution (0.9% NaCl, pH approximately 6). The alkaline medium was prepared by dissolving sodium bicarbonate (NaHCO_3_) in distilled water and adjusting the pH to 8.5.

The samples were immersed in the respective solutions and incubated for 2, 6 (corresponding to the manufacturer’s recommended replacement interval), and 10 weeks at 37 °C, in a laboratory oven AL01-02-100 (Advantage-Lab GmbH, Darmstadt, Germany). These incubation periods were selected to represent short-term exposure, clinically relevant routine use, and prolonged exposure, respectively. All samples were placed in sealed polypropylene containers with a liquid volume of 100 mL.

### 2.2. Mechanical Characterization

Mechanical properties were assessed using a standard static tensile test performed using an MTS Insight 50 universal testing machine equipped with Test Works v.4.0 software (MTS Systems Corporation, Eden Prairie, MN, USA). The tests were conducted at a constant crosshead speed of 50 mm/min. The initial gauge length was set to 100 mm. The specimens were tested in their original tubular geometry without flattening or modification prior to testing. The specimens were mounted using mechanical grips at both ends. Care was taken to ensure axial alignment and to minimize specimen slippage during testing. The cross-sectional area was determined as 11.74 mm^2^ for silicone and 9.02 mm^2^ for PVC specimens. Engineering strain was calculated from the crosshead displacement divided by the initial gauge length. Engineering stress was calculated as the applied force divided by the initial cross-sectional area of the specimen. Young’s modulus was estimated as the secant slope of the engineering stress–strain curve between 5% and 10% engineering strain:

(1) $$E=\frac{\sigma_{10}-\sigma_{5}}{\varepsilon_{10}-\varepsilon_{5}},$$

where $\sigma_{10}$ and $\sigma_{5}$ are the engineering stresses corresponding to 10% and 5% strain, respectively. The selected strain interval was used to provide a consistent basis for comparison of the investigated materials. Because strain was calculated from crosshead displacement rather than direct local strain measurements, the reported Young’s modulus values should be regarded as approximate estimates suitable for qualitative comparison. The tensile test was continued until specimen failure.

**Figure 1:**
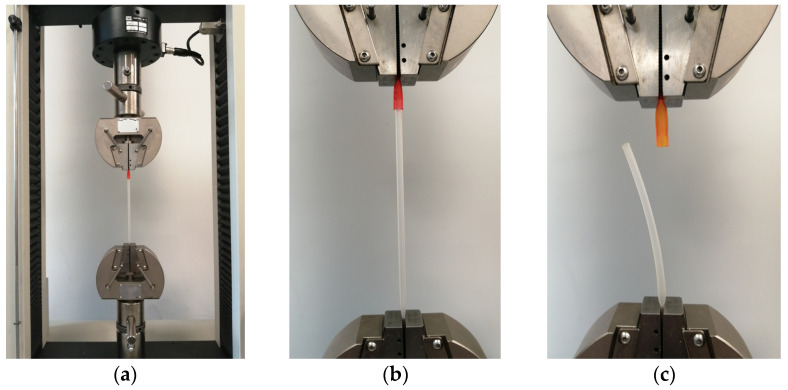
Representative photographs of the pull-off test: (**a**) overall experimental setup, (**b**) specimen mounting configuration, and (**c**) specimen after stopper detachment.

An additional pull-off test of the stopper was carried out to evaluate the attachment strength. The samples were mounted in a universal testing machine, and the stopper was pulled off along the axial direction at a constant crosshead speed of 50 mm/min until detachment. The maximum force (Fmax) was recorded for each measurement. Due to the limited number of samples (*n* = 2), mechanical results were interpreted qualitatively. Representative photographs illustrating the pull-off testing procedure and specimen configuration are shown in Figure 1.

### 2.3. Wettability and Surface Free Energy Measurements

Wettability was investigated using the sessile drop method with an optical goniometer (Advex Instruments) equipped with See System software v.6.3 (Advex Instruments, Brno, Czech Republic). The following probe liquids were distilled water (BIOSPACE, Poznań, Poland; ρ = 1 g/cm^3^), glycerol (Chempur, Piekary Śląskie, Poland; ρ = 1.26 g/cm^3^), and α-bromonaphthalene (Sigma-Aldrich, Merck Life Science, St. Louis, MO, USA; ρ = 1.48 g/cm^3^).

Droplets with a volume of 0.5 µL were dispensed using an OPTIPETTE OP10 micropipette (HTL, Warsaw, Poland). The surface free energy (SFE) was calculated based on the measured contact angle (θ). Contact angle measurements were performed directly on the original cylindrical tube surfaces. The tubes were neither cut nor flattened prior to testing to preserve their native surface characteristics. A droplet volume of 0.5 µL was selected to ensure stable droplet deposition on the curved surface and to minimize the influence of surface curvature on contact angle determination. No curvature correction was applied during image analysis. For each experimental condition, two specimens were analyzed. Four droplets were deposited at different locations on each specimen surface, resulting in a total of eight contact-angle measurements per condition. The specimen was considered the experimental unit, whereas the individual droplet measurements represented repeated technical measurements performed on the same specimen. Contact-angle data are presented as mean ± standard deviation (SD). The surface tension parameters of the probe liquids were taken from the database implemented in See System software v.6.3 (Advex Instruments, Brno, Czech Republic) and are summarized in Table 2.

The SFE ($\gamma_{S}$) of the tested gastric tube was determined using three approaches: Owens–Wendt–Rabel–Kaelble (OWRK), Wu, and van Oss–Chaudhury–Good (vOCG) methods. For the OWRK and Wu models, the dispersive and polar components were obtained from the liquid surface tension parameters available in the software database.

The OWRK model applies a geometric mean to describe the interactions between the polar ($\gamma_{L}^{p}$) and dispersive ($\gamma_{L}^{d}$) components of the liquid and solid surface energies:

(2) $$\left(1+cos\theta \right)\gamma_{L}=2\left(\sqrt{\gamma_{S}^{d}\gamma_{L}^{d}}+\sqrt{\gamma_{S}^{p}\gamma_{L}^{p}}\right),$$

(3) $$\gamma_{S}=\gamma_{S}^{d}+\gamma_{S}^{p},$$

where $\gamma_{S}^{d}$ and $\gamma_{S}^{p}$ denote the dispersive component (Lifshitz–van der Waals) and polar component of solids, respectively. A minimum of two probe liquids (polar and non-polar) is required [23].

The Wu method employs a harmonic mean [24]:

(4) $$\left(1+cos\theta \right)\gamma_{L}=4\left(\frac{\gamma_{S}^{d}\gamma_{L}^{d}}{\gamma_{S}^{d}+\gamma_{L}^{d}}+\frac{\gamma_{S}^{p}\gamma_{L}^{p}}{\gamma_{S}^{p}+\gamma_{L}^{p}}\right).$$

Two probe liquids (one polar and one non-polar) are used to measure the contact angle and determine the dispersive and polar components of the surface free energy.

The vOCG approach provides a more detailed description of intermolecular interactions by separating the surface free energy into Lifshitz-van der Waals ($\gamma_{S}^{LW}$) and acid–base component ($\gamma_{S}^{AB}$) components, the latter divided into two parts, acid ($\gamma_{S}^{+}$) and basic ($\gamma_{S}^{-}$) [25].

(5) $$\gamma_{S}=\gamma_{S}^{LW}+\gamma_{S}^{AB},$$

(6) $$\gamma_{S}^{AB}=2\sqrt{\gamma_{S}^{+}\gamma_{S}^{-}},$$

(7) $$\left(1+cos\theta \right)\gamma_{L}=2\left(\sqrt{\gamma_{S}^{LW}\gamma_{L}^{LW}}+\sqrt{\gamma_{S}^{+}\gamma_{L}^{-}}+\sqrt{\gamma_{S}^{-}\gamma_{L}^{+}}\right).$$

The combined use of the OWRK, Wu, and vOCG methods provides a comprehensive characterization of surface properties because the three approaches offer complementary descriptions of interfacial interactions. OWRK and Wu separate dispersive and polar contributions using different mathematical formalisms, whereas the vOCG approach additionally resolves acid–base interactions. Following incubation, specimens were first subjected to mass measurements and contact-angle analysis. Mechanical testing was subsequently performed as the final characterization step because it was destructive.

### 2.4. Mass Measurements

The mass of the samples was measured before incubation and after selected incubation periods (2, 6, and 10 weeks) using an analytical balance (Radwag AS 160, Radwag, Poland; readability 0.1 mg, maximum capacity 160 g). Each sample was gently dried prior to weighing. Due to the limited number of measurements (*n* = 2), the results were evaluated qualitatively.

### 2.5. Statistical Analysis

Because only two specimens were available for each condition and droplet measurements represented repeated technical measurements, the statistical analyses should be regarded as exploratory assessments of potential trends rather than formal hypothesis testing. An exploratory factorial analysis of variance (ANOVA) was performed separately for each probe liquid (distilled water, glycerol, and α-bromonaphthalene).

The statistical model included main effects of material type, incubation time, and pH, together with material × time interaction effects. The same approach was applied to surface free energy parameters determined using the OWRK, Wu, and vOCG methods. Contact-angle data are reported as mean ± standard deviation (SD) based on eight droplet measurements obtained from two specimens per experimental condition. For the purpose of the exploratory analyses, *p*-values below 0.05 were considered indicative of potential differences.

## 3. Results

### 3.1. Mechanical Characterization

**Figure 2:**
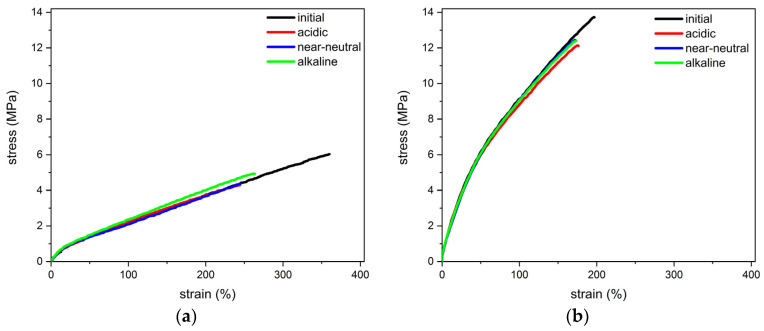
Representative stress–strain curves obtained after 10 weeks of incubation under different pH conditions: (**a**) silicone and (**b**) PVC.

The results of tensile testing of silicone and PVC gastric feeding tube samples exposed to controlled pH environments are presented in Figure 2.

As shown in Figure 2, the mechanical response of both materials is influenced by environmental conditions (acidic, neutral, and alkaline), although the extent of this influence differs between silicone and PVC. Silicone exhibits relatively low stress values, with a maximum stress of approximately 6 MPa and elongation reaching approximately 360%. The Young’s modulus is in the range of approximately 3–6 MPa. The stress–strain response displays quasi-linear behavior over a wide strain range. Differences in stress levels are observed depending on the environment, with the largest variability under alkaline conditions. However, the overall shape of the stress–strain curves remains consistent across all tested conditions, indicating that the deformation response is similar and that environmental effects are primarily reflected in stress magnitude, rather than in changes in deformation behavior. In contrast, PVC demonstrates higher strength and stiffness, reaching a maximum stress of approximately 11–14 MPa at a strain of about 160–210%. The Young’s modulus is approximately 13–16 MPa. The stress–strain curves also exhibit quasi-linear behavior and remain closely grouped for all environmental conditions. Only minor decreases in stress values are observed, most notably under acidic conditions. The similarity in the curves suggests a relatively stable mechanical response of PVC under varying pH conditions. Overall, PVC tended to show lower variability in mechanical response than silicone under the tested conditions; however, due to the limited number of specimens, these observations should be interpreted as qualitative trends. Detailed individual mechanical test results are provided in the Supplementary Material (Data S1), while the complete stress–strain and pull-off load–displacement curves are presented in Supplementary Figures S1 and S2.

**Figure 3:**
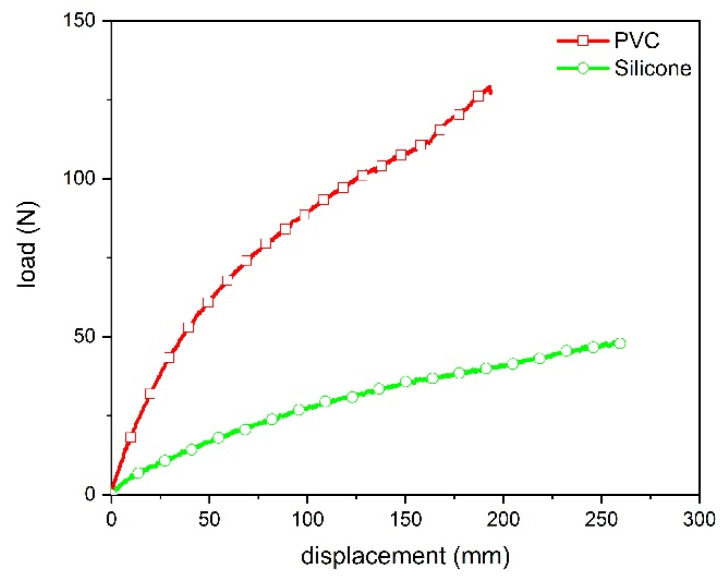
Load–displacement curves for the pull-off test of caps from gastric feeding tubes made of PVC and silicone.

Pull-off tests (Figure 3) revealed a substantially higher detachment force for PVC samples (approximately 130 N) compared to silicone (approximately 50 N). Although based on a limited number of measurements, the results consistently indicate stronger stopper attachment in PVC tubes.

### 3.2. Wettability and Surface Free Energy

The results correspond to different incubation times (0, 2, 6, and 10 weeks) and pH conditions (2, 6, and 8.5), with each value representing a specific combination of these variables. The contact angle measurements revealed distinct differences between PVC and silicone gastric feeding tubes, as well as the effects of incubation time and pH (Table 3).

For PVC surface, the contact angle values for water remained relatively stable, ranging from approximately 73° to 84°, indicating a moderately hydrophobic behavior. The contact angles for α-bromonaphthalene were consistently low (28–34°), confirming good wettability by a non-polar liquid. Glycerol showed greater variability, with values ranging from approximately 67° to 84°, depending on incubation conditions. For silicone, significantly higher water contact angles were observed (approximately 76° to 105°), indicating hydrophobic to highly hydrophobic behavior and lower wettability compared to PVC. The contact angles for α-bromonaphthalene ranged from 55° to 72°, while glycerol values remained relatively high (79–96°). Changes in wettability with incubation time were more pronounced for silicone than for PVC. For silicone, a general decrease in water contact angle was observed after 6 weeks of incubation, followed by partial recovery after 10 weeks, although the extent of these changes depended on the environmental conditions. The influence of pH was less consistent, and no clear trend was observed.

The surface free energy (SFE) values determined by the OWRK method showed clear differences between PVC and silicone gastric feeding tubes, as well as variations depending on incubation time and pH conditions (Table 4).

The OWRK analysis revealed relatively stable surface free energy values for PVC, with the total energy remaining within a narrow range of approximately 41–45 mJ/m^2^. The dispersive component dominated and showed minimal variation, whereas the polar component remained low but increased under certain conditions, particularly during prolonged incubation and in selected pH environments. In contrast, silicone exhibited pronounced changes during incubation. A marked increase in total surface free energy was observed after 6 weeks (up to ~33–34 mJ/m^2^), primarily due to a significant rise in the polar component, which reached ~10.54 mJ/m^2^ before decreasing again under selected conditions after prolonged incubation.

The surface free energy determined by the Wu method showed clear differences between PVC and silicone and varied with incubation conditions (Table 5).

The Wu method resulted in higher values of total surface free energy compared to the OWRK approach, particularly for PVC, where $\gamma_{S}$ ranged from approximately 45 to 51 mJ/m^2^. These values were consistently dominated by the dispersive component, while the polar contribution remained elevated (8–12 mJ/m^2^) relative to the OWRK model. Silicone showed a different trend, with initially low values (~26.5 mJ/m^2^) followed by a pronounced increase after 6 weeks of incubation (~39–40 mJ/m^2^). This change was mainly associated with an increase in the polar component, which reached up to ~15 mJ/m^2^ before decreasing again under selected conditions after prolonged incubation.

The surface free energy determined by the vOCG approach differed between PVC and silicone and varied with incubation conditions, as shown in Table 6.

The vOCG analysis highlighted pronounced variability in the acid–base components of surface free energy, particularly for silicone samples. While the total surface free energy of PVC remained relatively stable (~38–49 mJ/m^2^), changes in surface properties were mainly reflected in variations in the polar and acid–base parameters. Variations were observed in both the acid ($\gamma_{S}^{+}$) and base ($\gamma_{S}^{-}$) parameters during incubation, indicating changes in surface interaction properties.

### 3.3. Mass Changes

Relative mass changes remained low throughout the incubation period. Silicone exhibited variations between −2.0% and +1.1%, whereas PVC showed changes ranging from −0.7% to +2.5% (Table 7). Both increases and decreases in mass were observed depending on the incubation condition, and no consistent trend with respect to pH or exposure time was identified. Due to the limited number of specimens, these observations were interpreted descriptively.

### 3.4. Statistical Analysis

Descriptive statistical assessment suggested that material type was associated with differences in contact angle values for all tested liquids (*p* < 0.001). Incubation time appeared to influence contact angles measured with water and glycerol (*p* < 0.05–0.001), whereas the effect of pH was less consistent and reached statistical significance only in selected cases. Interactions between material type and incubation time (*p* < 0.01) were also observed, suggesting different surface modification behavior of the tested materials.

Exploratory analysis of variance applied to surface free energy values determined using the OWRK and Wu methods suggested that material type was associated with differences in total surface free energy (*p* < 0.001). Incubation time primarily affected the polar components, while the dispersive component remained relatively unchanged. Due to the high variability and complex nature of the acid–base components determined using the vOCG approach, these results were interpreted qualitatively in terms of general trends rather than statistically differentiated groups.

## 4. Discussion

The present findings complement the available literature by providing a combined assessment of the mechanical and surface response of gastric feeding tube materials under controlled pH conditions. Surface properties govern wettability, biocompatibility, and interactions with biological fluids [26]. Prolonged exposure to variable pH conditions may therefore impact both bulk and surface characteristics, influencing both mechanical response and interfacial behavior. PVC showed stable SFE values (~41–45 mJ/m^2^), whereas silicone exhibited a wider variation (~19–34 mJ/m^2^). Although the three methods yielded different absolute values, they showed similar qualitative trends. The OWRK model was considered the primary basis for interpretation because of its widespread use in polymer surface characterization and the relatively stable behavior of the calculated components. The differences between silicone and PVC can be explained by their distinct deformation mechanisms. Silicone, as a crosslinked elastomer, exhibits entropic elasticity associated with the stretching and reorientation of polymer chains, resulting in low stiffness and large reversible deformations. The greater surface sensitivity observed for silicone is consistent with previous reports showing that silicone-based feeding tubes may undergo surface modifications during prolonged exposure to physiological environments [15,27]. However, unlike studies reporting pronounced material degradation, the present work primarily identified changes in wettability and surface free energy rather than clear evidence of bulk material deterioration. The observed trends suggest that environmental conditions may influence stress levels, particularly under alkaline conditions, possibly due to increased chain mobility or mild swelling. Water uptake is a potential mechanism contributing to these observations, as prolonged exposure to aqueous environments may induce volumetric expansion and alter chain mobility in elastomeric materials. Although swelling behavior and chemical composition were not directly characterized in the present study, the consistent shape of the stress–strain curves suggests that the underlying deformation mechanism remains unchanged. Instead, environmental exposure appears to primarily influence the magnitude of the mechanical response rather than its fundamental nature. From a practical perspective, these differences in stiffness may influence insertion and positioning, as softer tubes are associated with improved positioning accuracy but may be more difficult to control during application [3,27,28].

In contrast, plasticized PVC behaves as an amorphous thermoplastic, where deformation is governed by intermolecular interactions and plasticizer-assisted chain mobility. The mechanical response combines elastic deformation and viscoplastic flow. The limited variation observed across different environments indicates that PVC exhibits relatively stable mechanical behavior, with changes mainly affecting stress magnitude rather than deformation mechanism. This stability may be attributed to the presence of plasticizers, which maintain chain mobility even under varying chemical conditions. In addition, prolonged exposure to aqueous environments may promote limited plasticizer migration or extraction, which has been reported to influence the mechanical and surface properties of plasticized PVC materials. Nevertheless, no chemical analyses were performed; therefore, the occurrence of plasticizer leaching could not be verified and remains speculative in the context of the present study. Available literature on PVC feeding tubes exposed to simulated gastric environments remains limited, with most studies focusing on silicone or polyurethane-based materials. Previous studies on feeding tube materials exposed to gastric environments have reported gradual degradation, changes in surface characteristics, and partial loss of mechanical properties, particularly for polyurethane-based devices [15,27]. Karov et al. [15] observed that exposure to simulated gastric conditions altered both the surface characteristics and the mechanical behavior of nasogastric feeding tubes. Similar observations were made in studies of polyurethane materials, where prolonged exposure resulted in measurable changes in mechanical performance [27]. In the present study, changes in both mechanical and surface properties were also observed; however, PVC tended to maintain a more stable response across the investigated conditions, whereas silicone exhibited greater variability, particularly in surface-related parameters. These findings are therefore generally consistent with previous reports indicating that environmental exposure may affect feeding tube materials, although the magnitude and nature of the observed changes appear to depend strongly on material composition. These observations are consistent with clinical studies reporting differences in performance and durability between silicone and other polymer-based feeding tubes [3,27]. From a clinical perspective, both excessively high and excessively low attachment forces may be undesirable. A stronger attachment may reduce the risk of accidental disconnection, whereas lower attachment forces may facilitate manipulation and removal. Since no functional clinical tests were performed, the present results should not be interpreted as evidence of superior clinical performance.

Changes were observed in both mechanical and surface characteristics; the present data do not permit conclusions regarding a direct connection between them, as correlations between the measured parameters were not evaluated. PVC tended to exhibit greater stability in both mechanical and surface properties, whereas silicone appeared to be more sensitive, particularly at the surface level.

Despite the valuable insights provided by this study, several limitations should be acknowledged. The mechanical tests were performed on a limited number of samples, and therefore the results should be interpreted primarily in terms of observed trends rather than precise quantitative relationships. Similarly, the statistical analyses of contact-angle and surface free energy measurements were based on repeated technical measurements obtained from a limited number of specimens and should therefore be interpreted as indicative of potential trends rather than statistically validated differences. For this reason, the observed differences between PVC and silicone should be regarded as indicative rather than statistically validated. In addition, the study was conducted under controlled in vitro conditions, which may not fully replicate the complexity of the in vivo environment, including the presence of biological agents, dynamic fluid interactions, and physiological mechanical loading encountered during clinical use.

Similar degradation phenomena in polymer-based medical tubes, including surface wear and material aging, have been reported in long-term studies under physiological conditions [15,29,30]. Nevertheless, the applied methodology enables a consistent comparison of material behavior and provides a useful basis for further investigations.

It should be noted that contact angle measurements performed on cylindrical medical devices may be affected by local surface curvature. Although a small droplet volume was used to minimize this effect, some influence of specimen geometry on the measured values cannot be completely excluded.

Since no complementary chemical or morphological analyses (e.g., FTIR, SEM, AFM, XPS, or profilometry) were carried out, the processes responsible for the observed surface modifications could not be identified. Therefore, no direct correlation between chemical or morphological changes and the observed mechanical and surface properties could be established. The partial recovery of contact angle values observed for silicone after prolonged incubation should therefore be interpreted cautiously, as the underlying mechanism cannot be determined without complementary surface characterization techniques. This behavior may reflect surface rearrangement processes, experimental variability, or other physicochemical changes; however, the available data do not allow discrimination between these possibilities. In addition to surface chemistry, wettability may also be influenced by surface topography and microstructural features [31]. Previous studies have demonstrated that micro- and nanoscale surface texturing can substantially modify the wetting behavior of polymeric materials, even in the absence of significant changes in surface chemistry [31]. Therefore, the contribution of topographical effects to the observed changes cannot be excluded and should be investigated in future studies.

It should also be noted that the incubation media were simplified aqueous solutions designed to provide controlled pH conditions. Thus, they do not reproduce the full chemical complexity of gastric fluids, including enzymes, proteins, electrolytes, food residues, bile components, and other biological factors that may affect material degradation in vivo. In addition, the experimental setup did not account for dynamic flow conditions or cyclic mechanical loading that may occur during clinical use.

Mass measurements were performed on only two specimens per condition. Therefore, the observed mass changes should be interpreted with caution and not as definitive evidence of the presence or absence of material degradation. Rather, the results indicate that no consistent trends related to pH or incubation time were detected under the present experimental conditions. The occurrence of both positive and negative mass changes suggests that the observed variations were more likely associated with minor liquid uptake and release phenomena, experimental variability, or specimen-to-specimen differences than with systematic material degradation.

From a practical perspective, the observed changes in mechanical and surface properties may be relevant to the long-term performance of feeding tubes. Variations in stiffness and attachment force may influence handling, positioning, and resistance to accidental disconnection, whereas changes in wettability and surface free energy may affect interactions with biological fluids. Although the observed modifications do not permit direct conclusions regarding device service life or replacement intervals, they suggest that prolonged environmental exposure may alter material performance over time. Hence, both mechanical and surface stability should be considered when evaluating the long-term reliability of feeding tube materials.

The target pH values of the incubation media were established during preparation; however, pH was not continuously monitored throughout the incubation period using a calibrated pH meter. Therefore, possible pH fluctuations between medium replacements cannot be excluded. Engineering strain was calculated from crosshead displacement relative to the initial gauge length. As a result, the reported Young’s modulus values should be considered approximate and are intended primarily for qualitative comparison between the investigated materials and conditions.

These findings highlight the fundamental role of surface properties, particularly wettability and surface free energy, in governing interactions between feeding tube materials and the biological environment [11,12]. Previous studies have demonstrated that bacterial adhesion to polymeric medical devices is influenced by surface physicochemical properties, including wettability, surface free energy, and topography [11,12,13,14]. In particular, Lima et al. [13] reported that the hydrophobicity and roughness of nasoenteral tube surfaces affected the adhesion of Staphylococcus aureus, while Vongbhavit et al. [14] demonstrated bacterial colonization of enteral feeding tubes under clinical conditions. The observed surface modifications may therefore be relevant to fluid interactions, bacterial adhesion, and biofilm formation. However, these effects were not directly investigated in the present study, and no microbiological testing was performed [29,30]. Consequently, no conclusions regarding microbial colonization or biofilm development can be drawn from the present data.

## 5. Conclusions

This study provides a combined evaluation of mechanical and surface properties of gastric feeding tubes under varying pH conditions. The results suggest that tube performance may depend on both material type and environmental exposure; however, mechanical observations should be interpreted qualitatively due to the limited number of specimens. Poly(vinyl chloride) (PVC) tended to exhibit higher stiffness and a more consistent mechanical response under the tested conditions. In contrast, silicone appeared to exhibit lower stiffness and greater sensitivity to environmental factors, with noticeable variations in stress levels despite maintaining a consistent deformation mechanism.

Surface analysis revealed that PVC maintained relatively stable wettability and surface free energy, with only minor changes primarily in the polar component. Conversely, silicone exhibited pronounced time-dependent surface modifications, particularly after prolonged incubation, reflected in significant variations in polar and acid–base components. These changes occurred alongside alterations in mechanical response. The observed variations in surface properties may influence material–environment interactions under clinical conditions. However, no microbiological testing was performed in the present study; therefore, the potential implications for bacterial adhesion and biofilm formation remain speculative.

## Figures and Tables

**Table 1:** Allocation of specimens used in the study.

Group	pH	Number of Specimens
Reference	Initial (0 weeks)	2
Acidic	pH 2	6
Near-neutral	pH 6	6
Alkaline	pH 8.5	6

**Table 2:** Surface tension parameters (mJ/m^2^) of probe liquids used in the OWRK, Wu, and vOCG calculations.

Probe Liquid	$\gamma_{L}$	$\gamma^{LW}$	$\gamma^{AB}$	$\gamma^{+}$	$\gamma^{-}$
Water	72.8	21.8	51.0	25.5	25.5
Glycerol	64.0	34.0	30.0	3.9	57.4
α-Bromonaphthalene	44.4	44.4	0	0	0

**Table 3:** Contact angle values (°) for PVC and silicone gastric feeding tubes measured with distilled water, α-bromonaphthalene, and glycerol after incubation under different pH conditions and time periods (mean ± SD).

Time/pH	PVC			Silicone		
	DW	αB	G	DW	αB	G
0 (initial)	80.33 (6.39) ^b^	34.56 (2.71) ^a^	70.14 (2.98) ^ab^	103.00 (2.53) ^a^	68.54 (7.21) ^c^	83.59 (9.10) ^bc^
2/2	79.99 (4.80) ^b^	29.94 (3.90) ^a^	69.41 (9.04) ^ab^	95.75 (4.82) ^ab^	68.36 (5.93) ^c^	95.84 (5.50) ^a^
2/6	78.35 (3.55) ^b^	28.62 (9.22) ^a^	68.56 (14.62) ^ab^	105.45 (2.66) ^a^	66.99 (3.67) ^c^	87.37 (7.25) ^bc^
2/8.5	81.23 (3.91) ^b^	30.79 (3.62) ^a^	77.96 (8.04) ^b^	103.87 (2.84) ^a^	59.99 (5.49) ^c^	84.95 (9.25) ^bc^
6/2	82.71 (4.22) ^b^	28.05 (3.50) ^a^	66.94 (4.42) ^a^	76.61 (1.95) ^c^	66.52 (4.68) ^bc^	79.07 (2.07) ^ab^
6/6	83.81 (2.83) ^ab^	33.26 (5.48) ^ab^	76.02 (3.39) ^b^	76.97 (2.39) ^c^	61.28 (3.92) ^bc^	85.88 (11.12) ^ab^
6/8.5	73.64 (3.14) ^c^	32.76 (4.94) ^ab^	72.18 (5.93) ^ab^	80.41 (1.61) ^c^	54.95 (2.15) ^b^	84.67 (6.77) ^ab^
10/2	73.98 (4.92) ^c^	28.61 (4.70) ^a^	75.39 (2.76) ^b^	85.49 (4.66) ^bc^	68.35 (5.52) ^bc^	83.16 (2.95) ^ab^
10/6	76.35 (5.21) ^bc^	31.93 (2.71) ^ab^	83.90 (2.90) ^c^	103.75 (7.19) ^a^	71.80 (5.50) ^c^	87.43 (7.48) ^bc^
10/8.5	82.42 (2.41) ^b^	28.15 (1.83) ^a^	73.42 (3.39) ^ab^	97.92 (5.36) ^ab^	56.54 (7.51) ^b^	87.58 (8.46) ^bc^

Note: DW—distilled water, αB—α-bromonaphthalene, G—glycerol. Different letters indicate groupings obtained from the exploratory statistical analysis. Because the measurements represent repeated technical measurements rather than independent biological replicates, these groupings should be interpreted as indicative trends rather than statistically validated differences.

**Table 4:** Surface free energy (mJ/m^2^) and its components determined by the Owens–Wendt–Rabel–Kaelble (OWRK) method for PVC and silicone gastric feeding tubes after incubation (mean ± SD).

Time/pH	PVC			Silicone		
	$\gamma_{S}$	$\gamma_{S}^{d}$	$\gamma_{S}^{p}$	$\gamma_{S}$	$\gamma_{S}^{d}$	$\gamma_{S}^{p}$
0 (initial)	41.08 (2.81) ^b^	36.88 (1.10) ^a^	4.19 (2.29) ^b^	21.85 (3.08) ^d^	20.78 (3.51) ^c^	1.08 (0.62) ^c^
2/2	42.68 (0.98) ^b^	38.71 (1.68) ^a^	3.97 (1.05) ^b^	23.74 (2.12) ^c^	20.84 (2.9) ^c^	2.89 (1.79) ^b^
2/6	42.39 (1.11) ^b^	38.65 (0.81) ^a^	3.74 (1.00) ^b^	22.06 (1.69) ^d^	21.49 (1.82) ^c^	0.57 (0.43) ^c^
2/8.5	41.74 (1.72) ^b^	38.31 (1.25) ^a^	3.43 (1.23) ^b^	25.45 (2.70) ^c^	24.98 (2.75) ^b^	0.47 (0.31) ^c^
6/2	40.11 (1.99) ^b^	37.32 (2.02) ^a^	2.79 (0.77) ^c^	32.27 (1.15) ^a^	21.73 (2.32) ^c^	10.54 (2.00) ^a^
6/6	42.14 (0.93) ^b^	39.29 (1.13) ^a^	2.86 (1.22) ^c^	33.52 (1.88) ^a^	24.34 (1.97) ^b^	9.19 (1.34) ^a^
6/8.5	43.97 (2.11) ^ab^	37.53 (1.76) ^a^	6.44 (1.17) ^a^	33.88 (0.73) ^a^	27.51 (1.08) ^b^	0.80 (0.26) ^c^
10/2	43.27 (2.05) ^ab^	37.90 (1.02) ^a^	5.37 (1.98) ^ab^	27.35 (2.96) ^c^	20.84 (2.72) ^c^	6.51 (2.09) ^b^
10/6	45.05 (2.11) ^a^	39.05 (1.55) ^a^	6.00 (2.17) ^a^	19.15 (1.78) ^d^	18.35 (1.92) ^d^	0.80 (0.26) ^c^
10/8.5	42.11 (1.05) ^b^	39.29 (0.58) ^a^	2.82 (0.61) ^b^	27.83 (4.09) ^c^	26.68 (3.71) ^b^	1.15 (0.63) ^c^

Note: Interpretation of letter groupings is the same as described in Table 3.

**Table 5:** Surface free energy (mJ/m^2^) and its components determined by the Wu method for PVC and silicone gastric feeding tubes after incubation under different pH conditions and time periods (mean ± SD).

Time/pH	PVC			Silicone		
	$\gamma_{S}$	$\gamma_{S}^{d}$	$\gamma_{S}^{p}$	$\gamma_{S}$	$\gamma_{S}^{d}$	$\gamma_{S}^{p}$
0 (initial)	46.85 (3.67) ^b^	37.19 (1.08) ^a^	9.66 (3.14) ^b^	26.54 (2.52) ^b^	23.10 (3.18) ^b^	3.45 (1.27) ^b^
2/2	48.24 (1.49) ^b^	38.83 (0.82) ^a^	9.41 (1.37) ^b^	29.44 (2.19) ^b^	23.15 (2.62) ^b^	6.28 (2.55) ^b^
2/6	48.62 (1.01) ^b^	38.90 (1.69) ^a^	9.72 (1.36) ^b^	26.10 (1.68) ^b^	23.7 (1.65) ^b^	2.40 (1.02) ^b^
2/8.5	47.44 (2.23) ^b^	38.52 (1.25) ^a^	8.92 (1.78) ^b^	28.91 (2.59) ^b^	26.67 (2.51) ^b^	2.24 (1.27) ^b^
6/2	47.55 (1.45) ^b^	39.43 (1.15) ^a^	8.12 (1.96) ^b^	39.04 (1.16) ^a^	23.90 (2.10) ^b^	15.14 (1.22) ^a^
6/6	45.53 (2.24) ^b^	37.60 (2.00) ^a^	7.93 (1.21) ^b^	40.21 (1.39) ^a^	26.11 (1.79) ^b^	14.10 (1.39) ^a^
6/8.5	49.39 (2.25) ^b^	37.86 (1.87) ^a^	11.53 (2.90) ^a^	40.13 (0.97) ^a^	28.43 (1.57) ^b^	11.70 (1.22) ^a^
10/2	51.48 (2.56) ^a^	39.22 (1.56) ^a^	12.26 (2.60) ^a^	33.99 (3.20) ^b^	23.15 (2.45) ^b^	10.84 (2.37) ^b^
10/6	49.46 (2.70) ^b^	38.13 (1.02) ^a^	11.33 (2.68) ^a^	23.89 (1.62) ^b^	21.04 (1.73) ^b^	2.85 (0.53) ^b^
10/8.5	47.63 (1.43) ^b^	39.43 (0.58) ^a^	8.20 (1.00) ^b^	32.26 (4.64) ^b^	28.14 (3.43) ^b^	4.12 (1.58) ^b^

Note: Interpretation of letter groupings is the same as described in Table 3.

**Table 6:** Surface free energy (mJ/m^2^) and its components determined by the van Oss–Chaudhury–Good (vOCG) method for PVC and silicone gastric feeding tubes after incubation under different pH conditions and time periods (mean ± SD).

Time/pH	PVC					Silicone				
	$\gamma_{S}$	$\gamma_{S}^{LW}$	$\gamma_{S}^{AB}$	$\gamma^{+}$	$\gamma^{-}$	$\gamma_{S}$	$\gamma_{S}^{LW}$	$\gamma_{S}^{AB}$	$\gamma^{+}$	$\gamma^{-}$
0 (initial)	38.16 (0.87)	36.88 (1.00)	1.28 (0.33)	0.36 (0.35)	7.12 (5.37)	27.52 (6.91)	20.78 (2.95)	6.74 (4.29)	6.70 (1.66)	2.13 (1.79)
2/2	40.10 (1.51)	38.65 (1.15)	1.45 (1.15)	0.31 (0.31)	6.31 (2.51)	25.50 (4.31)	20.84 (2.68)	4.65 (2.57)	5.7 (1.72)	1.46 (1.23)
2/6	42.93 (3.50)	38.71 (1.68)	4.22 (2.33)	0.67 (0.40)	6.85 (4.52)	31.39 (4.04)	21.49 (1.61)	9.90 (2.75)	6.99 (0.96)	3.36 (1.29)
2/8.5	43.34 (3.66)	38.31 (1.01)	5.03 (3.86)	0.59 (0.22)	10.19 (4.64)	38.84 (5.72)	24.98 (2.43)	13.86 (4.64)	8.22 (1.33)	5.35 (2.86)
6/2	41.07 (1.12)	39.29 (1.13)	1.79 (0.91)	0.42 (0.36)	3.43 (2.30)	30.59 (1.84)	21.73 (1.97)	8.86 (0.29)	2.92 (0.77)	9.81 (3.62)
6/6	38.18 (2.46)	37.31 (2.03)	0.87 (0.05)	0.18 (0.05)	5.99 (1.22)	33.07 (1.72)	24.33 (1.74)	8.74 (0.88)	3.15 (0.72)	6.30 (2.09)
6/8.5	40.77 (1.92)	37.53 (1.77)	3.24 (1.59)	0.41 (0.14)	14.40 (3.28)	33.23 (0.80)	27.51 (0.80)	5.73 (1.17)	5.39 (0.53)	1.81 (0.84)
10/2	43.88 (2.95)	39.05 (1.55)	4.83 (2.35)	0.31 (0.26)	16.44 (5.71)	26.69 (2.70)	20.84 (2.42)	5.85 (1.54)	4.62 (0.94)	3.78 (2.48)
10/6	48.95 (5.26)	37.90 (1.02)	11.04 (5.24)	1.63 (1.63)	19.81 (5.24)	24.29 (3.14)	18.35 (1.48)	5.94 (1.87)	5.62 (0.76)	1.55 (0.71)
10/8.5	40.87 (1.51)	39.29 (0.58)	1.58 (1.24)	0.17 (0.17)	6.07 (2.51)	35.67 (4.57)	26.68 (2.30)	8.99 (1.93)	8.79 (1.11)	2.50 (1.01)

**Table 7:** Relative mass change (%) of PVC and silicone gastric feeding tubes after incubation under different pH conditions.

pH	PVC			Silicone		
	2	6	10	2	6	10
2	1.23	2.47	2.35	−1.96	−0.81	0.14
6	−0.70	0.88	1.64	−0.81	0.62	0.14
8.5	0.18	1.82	0.12	1.15	0.24	−1.05

## Data Availability

The original contributions presented in this study are included in the article and Supplementary Materials. Further inquiries can be directed to the corresponding author.

## Supplementary Materials

The following supporting information can be downloaded at: https://www.mdpi.com/article/10.3390/ma19153158/s1, Data S1: Experimental Results; Figure S1: Stress–strain curves for gastric feeding tubes under all investigated incubation conditions: (a) silicone, (b) PVC; Figure S2: Individual load–displacement curves obtained during the stopper pull-off tests of PVC and silicone gastric feeding tubes.
