# Supplementary material for: Mechanical Behavior and Surface Properties of Silicone and PVC Gastric Tubes Under Controlled pH Conditions
Source: Materials (Basel). 2026 Jul 23;19(15):3158. doi: 10.3390/ma19153158 (PMC13466749; doi:10.3390/ma19153158)
Supplement: Supplementary file 1 [file materials-19-03158-s001.zip › Figure S1.pdf]

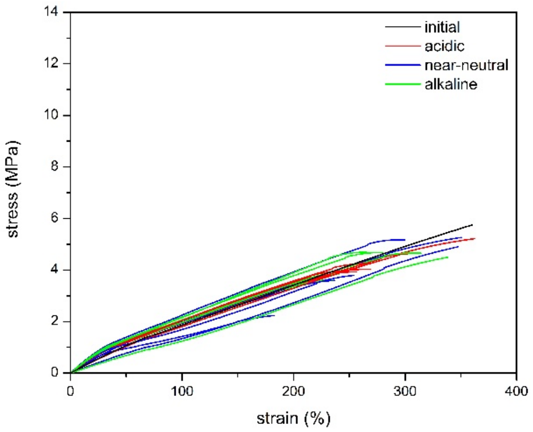

(a)

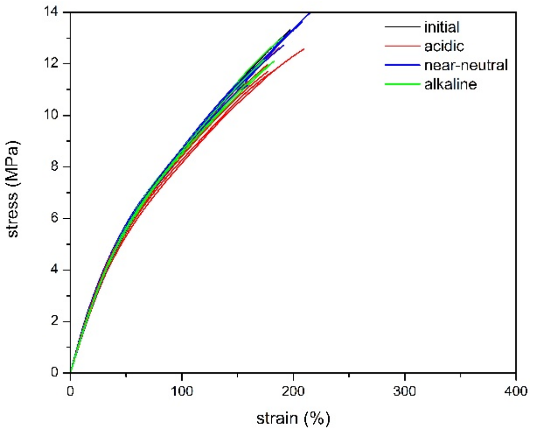

(b)

Figure S1. Stress–strain curves for gastric feeding tubes under all investigated incubation conditions: (a) silicone, (b) PVC.
