# Supplementary material for: Mechanical Behavior and Surface Properties of Silicone and PVC Gastric Tubes Under Controlled pH Conditions
Source: Materials (Basel). 2026 Jul 23;19(15):3158. doi: 10.3390/ma19153158 (PMC13466749; doi:10.3390/ma19153158)
Supplement: Supplementary file 1 [file materials-19-03158-s001.zip › Figure S2.pdf]

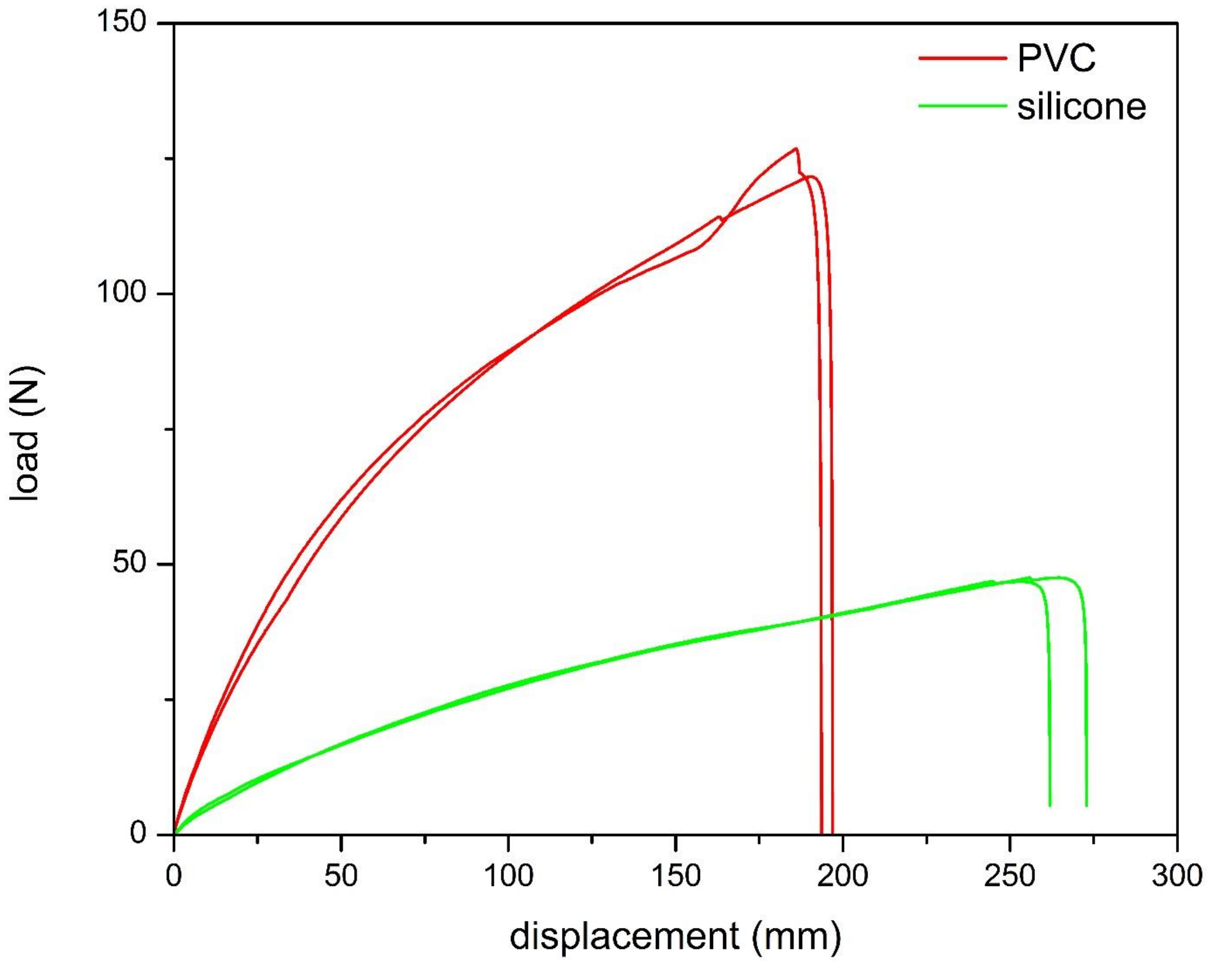

Figure S2. Individual load–displacement curves obtained during the stopper pull-off tests of PVC and silicone gastric feeding tubes.
